# Chronic recurrent multifocal osteomyelitis (CRMO): typical patterns of bone involvement on MRI with particular emphasis on Whole Body MRI (WBMRI)

**DOI:** 10.1186/1546-0096-13-S1-P53

**Published:** 2015-09-28

**Authors:** L Tanturri de Horatio, I Casazza, S Savelli, M Pardeo, V Messia, D Barbuti, P Tomà, F de Benedetti, A Insalaco

**Affiliations:** 1 Bambino Gesù Children Hospital, Department of Radiology, Rome, Italy; 2 Bambino Gesù Children Hospital, Pediatric Medicine-Rheumatology, Roma, Italy

## Introduction

Chronic recurrent multifocal osteomyelitis (CRMO) is an autoinflammatory bone disorder of unknown ethiology. The clinical manifestations of CRMO are highly variable. One to 20 sites can be affected at one time. Since CNO is a systemic disorder that can affect multiple skeletal sites, whole-body imaging techniques (Tc-99 bone scintigraphy or MRI) provide major contribution to the initial diagnostic approach, as well as during follow-up.

## Objectives

To evaluate typical patterns of bone involvement on MRI in paediatric patients with CRMO.

## Materials and methods

We retrospectively reviewed 112 MRI performed at the diagnosis and during follow-up from 2010 to 2014 of 40 children with CRMO. Thirty-two patients underwent bone biopsy that confirmed the diagnosis. 28/40 (70%) underwent one or more WBMRI. Coronal STIR images were obtained in all children. Additional sequences were performed in doubtful cases.

## Results

A total of 360 lesions were detected. Lesions were multifocal in 36/40 patients (90%) and were symmetric in at least one localization in 24/40 patients (60%). In 30/40 patients (75%) lesions were located on the metaphyses of long bones (especially femur and tibia) close to one or both sides of an epiphyseal or apophyseal growth plate and/or on pelvic bones (particularly on the sacro-iliac joints and close to the triradiate cartilage) and/or on clavicle/sternum. The spine was involved in 14/40 (34.1%) patients, in all but 2 in combination to the submentioned locations. No patients had carpal or head bone involvement, 16/40 had tarsal involvement.

## Conclusion

MRI, and particularly WBMRI, should be considered the diagnostic modality of choice in patients with clinical multifocal pain. Simmetricity, multifocality and particularly specific patterns of lesions appear highly suggestive of CRMO.

